# Role and Effective Therapeutic Target of Gut Microbiota in Heart Failure

**DOI:** 10.1155/2019/5164298

**Published:** 2019-11-16

**Authors:** Qiujin Jia, Hao Li, Huan Zhou, Xiaonan Zhang, Ao Zhang, Yingyu Xie, Yanyang Li, Shichao Lv, Junping Zhang

**Affiliations:** ^1^First Teaching Hospital of Tianjin University of Traditional Chinese Medicine, 314 An Shan Xi Road, Nan Kai District, Tianjin 300193, China; ^2^Affiliated Hospital of Nankai University, 4 Wei Shan Road, He Xi District, Tianjin 300222, China; ^3^Epidemiology, College of Global Public Health, New York University, 726 Broadway, New York, NY 10003, USA; ^4^Tianjin University of Traditional Chinese Medicine, 10 Po Yang Lake Road, Tuan Bo New Town West, Jing Hai District, Tianjin 301617, China; ^5^Tianjin Medical University Cancer Institute and Hospital, Tianjin 300193, China

## Abstract

Although the mechanism of the occurrence and development of heart failure has been continuously explored in the past ten years, the mortality and readmission rate of heart failure is still very high. Modern studies have shown that gut microbiota is associated with a variety of cardiovascular diseases, among which the study of gut microbiota and heart failure attracts particular attention. Therefore, understanding the role of gut microbiota in the occurrence and development of heart failure will help us further understand the pathogenesis of heart failure and provide new ideas for its treatment. This paper introduced intestinal flora and its metabolites, summarized the changes of intestinal flora in patients with heart failure, clarified that intestinal barrier damage and bacterial translocation induced inflammation and immune response aggravated heart failure, and altered intestinal microflora affected various metabolic pathways including trimethylamine/TMAO, SCFA, and Bile acid pathway leads to heart failure. At the same time, regulating intestinal microflora through diet, probiotics, antibiotics, fecal transplantation and microbial enzyme inhibitors has grown up to be a potential treatment for many metabolic disorders.

## 1. Introduction

Heart failure is a severe and terminal stage of many cardiovascular diseases and is an important part of the global prevention and treatment of chronic cardiovascular diseases. Epidemiological data show that the prevalence of heart failure in adults is 1% to 2% and increases to more than 10% of people over the age of 70 [1, 2]. With the ageing of the population, the incidence of chronic diseases such as coronary heart disease, hypertension, diabetes, obesity is on the rise, and the improvement of medical level, the survival time of patients with heart disease is prolonged, resulting in a continuous increase in the prevalence of heart failure. Heart failure is a difficult clinical syndrome caused by a variety of causes of abnormal changes in cardiac structure and function, resulting in ventricular systolic and/or diastolic function disorders [3]. Currently, heart failure is considered as a chronic, spontaneous and progressive disease, and the activation of the neuroendocrine system leads to pathological myocardial remodelling, which is the crucial factor in the occurrence and development of heart failure [4]. In the field of modern medical treatment, many drugs are being used, including beta-blockers, angiotensin-converting enzyme inhibitors and angiotensin receptor blockers (ARB), aldosterone antagonists, and combination of ARB/neprilysin blockers, ivabradine [5]. However, current treatments target only a fraction of the putative pathophysiological pathways, the overall prognosis of heart failure remains poor, readmission rates and mortality rates remain high, and even in the PARADIGM study, the 2-year mortality rate in the trial group was as high as 20% [6]. In addition, patients with heart failure are under a low quality of life, and long-term medication imposes a heavy financial burden on patients. Therefore, prevention of heart failure, timely diagnosis and early treatment are key to successful mortality reduction and prognosis. Gut microbiota is a unique ecosystem, and it functions as an endocrine organ, produces a plethora of metabolism dependent and metabolism-independent signals that play regulatory roles in cardiovascular disease development in the host [7]. More and more studies have shown that gut microbiota is closely related to the occurrence and development of heart failure, so microbiota is expected to become an essential target for intervention of heart failure.

## 2. Gut Microbiota and Its Metabolites

Intestinal micro-ecosystem is composed of gastrointestinal tract lumen, epithelial cell secretion, gut microbiota and substances entering the intestinal tract. Gut microbiota is the most important active ingredient in intestinal microecosystem [8]. The human body harbors 10–100 trillion microbes, mainly bacteria in our gut, which greatly outnumber our human cells [9]. The gut microbiota in the human body is mainly composed of *Bacteroides, Firmicutes, Actinobacteria,Proteobacteria *and* Verrucomicrobia. *Among them, *Firmicutes* and *Bacteroides* are dominant, accounting for more than 90% of the total intestinal microflora, and the remaining bacteria are less than 1% of the total gut microbiota [10, 11]. Owing to differences in host genes and external environmental factors (e.g., use of antibiotics, diet structure, lifestyle), the proportion of this flora is different in different individuals or different organs of the same individual [12, 13]. Flora can not only participate in the food digestion and nutrient uptake, providing energy for the host but also secrete metabolites, which can be viewed as hormone-like factors by dedicated receptor systems in the human host [14].

At present, gut microbiota interacts with the host through metabolism-independent pathways, such as lipopolysaccharide (LPS) and peptidoglycan, which are bacterial cell wall products, and metabolite-driven pathways, such as short-chain fatty acid (SCFA), trimethylamine (TMA)/trimethylamine N-oxide (TMAO) and bile acid (BA) [7]. Intestinal flora produces SCFAs by decomposing dietary fiber, mainly including acetic acid, propionic acid and butyric acid. The proportion of them in the colon is about 60 : 25 : 15 [15]. SCFAs can provide energy for intestinal epithelial cells and can also be involved in metabolic, immune and inflammatory responses as signaling molecules [16]. TMAO is only a little molecule compound. It is mainly transformed from choline, phosphatidylcholine and L-carnitine-rich foods (red meat, poultry, fish and eggs) by intestinal microorganisms to TMA, which are produced under the action of hepatic and flavin monooxygenase (FMO) [17]. BA is a vital part of bile. Primary bile acid changes into secondary bile acid through microbiota, and the composition changes of the bile acid pool can also affect the distribution of gut flora [18]. BAs can facilitate the absorption of dietary fat, fat-soluble molecules and cholesterol [19]. Besides, the gut microbiota is involved in the formation and regulation of the intestinal mucosal barrier [20], controlling nutrient intake, storage and metabolism [21], assisting the maturation of immune tissues, and preventing the growth of pathogenic microorganisms in the body [22]. Under physiological conditions, the intestinal flora is located in a state of balance. Once the balance is broken, pathogenic microorganisms thrive, leading to intestinal related diseases, such as inflammatory bowel disease, obesity, allergic diseases, diabetes, autism, colorectal cancer and cardiovascular diseases. In recent years, intestinal flora and cardiovascular diseases including coronary heart disease, hypertension, and heart failure have received sustained attention, and more and more evidence indicates that there is a close relationship between intestinal flora and heart failure [23–25].

## 3. Gut Microbiota Dysbiosis in Heart Failure

Exogenous factors such as diet, exposure to bacterial infections or taking drugs can reduce the diversity of intestinal flora; endogenous factors such as acute humoral imbalance, chronic intestinal congestion or ischemia-hypoxia, acid-base imbalance, weakened gastrointestinal motility, and nutritional deficiency can potentially change intestinal flora [26]. With the development of heart failure, the community characteristics of bacteria have changed. Studies have shown that the intestinal flora abundance of chronic heart failure patients decreased, and the number of pathogenic bacteria increased significantly with the progress of the disease, including *Campylobacter*, *Shigella*, *Salmonella*, *Yersinia enterocolitica* and *Candida species *[27]. According to Luedde et al., heart failure cases showed a significant decrease in *Coriobacteriaceae*, *Erysipelotrichaceae *and *Ruminococcaceae* at the family level and a significant decrease in *Blautia*, *Collinsella*, *unclassifified (uncl.) Erysipelotrichaceae *and *uncl. Ruminococcaceae* at the genus level [28]. A 16SrDNA analysis based on 22 hospitalized patients with heart failure reported a reduction in SCFA producing bacteria such as *Eubacterium rectale* and *Dorea longicatena* [29]. In addition, another study also shows that the gut microbiota signature in chronic heart failure is characterized by large compositional shifts with low bacterial richness and depletion of bacteria with butyrate-producing potential [30]. Butyrate exerts local anti-inflammatory effects in the gut mucosa and stimulates regulatory T-cells, also in the periphery [31]. Cui et al. observed that microbial genes for LPS biosynthesis and TMAO generation were up-regulated and genes for butyrate acetoacetate coenzyme A transferases (the key enzyme for the generation of butyrate) was down-regulated in chronic heart failure [25]. In conclusion, the intestinal flora of patients with heart failure changed, beneficial bacteria decreased, and pathogenic bacteria increased. The occurrence and development of heart failure may be linked to the decrease of SCFA-producing bacteria and the increase of TMAO-producing bacteria, which may become a new target for the treatment of heart failure.

## 4. The Role of Gut Microbiota in the Development of Heart Failure

### 4.1. Gut Barrier Dysfunction and Inflammation

Recently, more and more studies have confirmed that the intestinal tract plays an important role in the pathogenesis of heart failure, which is often referred to as the “gut hypothesis of heart failure”. The gut hypothesis implies that decreased cardiac output and redistribution of systemic circulation can lead to a decrease in intestinal perfusion and mucosal ischemia, which creates hypoxia and a hypercapnia status [32]. Subsequently, a decrease in intestinal mucosal PH and diminished activity of passive carrier-mediated transport occurs [32, 33], leading to a “leaky gut,” which describes increased gut permeability as well as intestinal barrier dysfunction. This disruption in intestinal barrier function, in turn, can lead to increased gut permeability, increased bacterial translocation and increased circulating endotoxins that can contribute to the underlying inflammation seen in patients with heart failure [32]. Sandek et al. proved that patients with chronic heart failure had increased thickness of the intestinal wall, intestinal permeability, and intestinal insufficiency [33]. A study also found that patients with moderate to severe congestive heart failure had increased intestinal permeability that observed a 78% increase via a sugar cellobiose test compared to the healthy controls, with a correlation between high right atrial pressure and increased intestinal permeability [27]. Another study by Sandek et al. showed that a higher concentration of juxta mucosal anaerobic bacteria in the sigmoid colon in patients was correlated with a higher systemic concentration of anti-LPS IgA antibodies and above a certain threshold, the more bacteria there are in the biofilm, the higher the LPS antibodies [34]. More definitive investigations have identified increased concentrations of endotoxin, specifically LPS, in edematous patients with heart failure [35]. Besides, during acute heart failure, a higher LPS concentration was found in the hepatic vein compared with the left ventricle, suggesting that bacteria migrated from the intestine to the systemic circulation [36]. The circulating endotoxins generated by bacteria refer to the main structural components of bacteria, including LPS, peptidoglycans, and so on. LPS and peptidoglycans interact with host mucosal surface cells through pattern recognition receptors such as toll-like receptors (TLRs) and nucleotide oligomerization domain-containing receptors (NODs) to recognize microbe-associated molecular patterns (MAMPs), stimulate and direct host immune responses [7, 37]. It is currently believed that low levels of gut-derived bacteria can appear in the circulation , leading to chronic low-grade inflammation, known as “metabolic endotoxemia”, which has been found in many chronic metabolic diseases such as obesity, type 2 diabetes and atherosclerosis [38]. Of course, this chronic low-grade inflammation can undoubtedly accelerate the development of heart failure. LPS-induced TLR4 activation induces the release of inflammatory cytokines like tumour necrosis factor-*α* (TNF-*α*), interleukin (IL)-1 and IL-6 [39]. However, these inflammatory mediators are associated with cardiac apoptosis, hypertrophy, and fibrosis [33]. Additionally, LPS itself can continue to promote deterioration of the mucosal barrier function. When LPS was up-regulated, the expression of ZO-1(Zonulaoccludens-1) and Occludin decreased [40, 41], and intestinal permeability increased. Therefore, the assessment of future intestinal barrier function may help us gain a better understanding of the occurrence and development of heart failure.

### 4.2. Intestinal Mucosal Immunity

Recent studies have shown that the immune activation mechanism marked by elevated inflammatory cytokines plays a vital role in the development of chronic heart failure. The interaction between gut microbiota and mucosal immunity is related to the occurrence of heart failure. Th17 cells are a subtype of CD_4_^+^ helper T cells, which is critical to the body to resist bacterial and fungal infections. Th17 cells play a role in the development of autoimmune diseases by secreting inflammatory factors such as IL-17, IL-22, IL-21 and recruiting neutrophils. Studies have demonstrated that sectional filamentous bacteria can promote the differentiation of Th17 cells in mice. The mechanism may be that sectional filamentous bacteria can induce the expression of serum amyloid A after colonisation into host epithelial cells, which can stimulate the secretion of IL-6 and IL-23 by dendritic cells in the lamina propria of the intestine, thereby promoting the differentiation of Th17 cells [42]. Clinical studies showed that Th17 cells in Acute Viral Myocarditis patients were hyperfunction and helped *B* cells to generate anti-myocardial antibodies [43]. Necessary studies have shown that IL-17 can promote myocardial inflammation and myocardial ischemia reperfusion injury [44, 45]. These results suggest that gut microbiota can encourage the development of chronic heart failure by influencing intestinal mucosal immunity. Polysaccharide A secreted by *Bacteroides fragilis* can induce CD_4_^+^ T cells to transform into Foxp^3+^ Treg cells, and Foxp^3+^ Treg cells can secrete anti-inflammatory factor IL-10 to regulate intestinal mucosal immune tolerance [46]. Treg cells control the abnormal expression of T cell receptors and CD_4_^+^ T cell proliferation, inhibit intestinal inflammation, and secrete inflammatory inhibitors TGF-*β* and IL-10, which mediate intestinal mucosal immune stability [47]. In addition, the research found that Treg cells can reduce ventricular remodelling after infarction by reducing apoptosis of myocardial cells and myocardial fibrosis [48]. Therefore, it is expected to improve heart failure by mediating intestinal mucosal immunity, thus providing a novel target for the treatment of heart failure.

### 4.3. The Gut Microbiota Metabolite-TMAO

Compared with patients with no heart failure, TMAO levels were higher in patients with chronic heart failure and associated with NYHA (New York Heart Association) grades, ischaemic aetiology and adverse outcomes [49]. A meta-analysis of 19 prospective studies in 19256 subjects showed that elevated plasma TMAO levels were associated with an increased relative risk of major adverse cardiovascular events, and the increase in relative risk did not change with BMI, diabetes, history of cardiovascular disease, renal dysfunction and other variables [50]. The TMAO level was significantly correlated with unfavourable outcomes (mortality and re-hospitalisation) in 2234, new or worsening heart failure patients, but guideline-based drug therapy did not affect the level of TMAO [51]. Besides, the study has shown that TMAO levels are associated with high BNP (B-type natriuretic peptide) levels and late left ventricular diastolic dysfunction, and can predict significant clinical adverse reactions for five years [52].

We have mentioned that TMAO levels are raised in chronic heart failure patients, but the mechanism of their increase is multifactorial. Changes in the composition of intestinal bacteria have turned out to be the principal drivers of TMAO levels [53]. Of course, TMAO levels are likewise primarily affected by TMAO substrate intakes, such as choline and betaine. Moreover, chronic heart failure patients have impaired intestinal mucosal barrier and increased permeability, which makes TMAO easier to enter the bloodstream through the intestinal mucosal barrier, leading to elevated levels. When mice with normal intestinal flora were fed a choline-rich diet, circulating TMAO levels increased, causing foam cell aggregation and promoting atherosclerotic plaque formation [17]. Mechanistic studies demonstrate that TMAO acts to potentiate platelet reactivity through alterations in stimulus-dependent calcium signaling [54]. Therefore, TMAO can increase atherosclerosis and thrombosis, which are entwined in the upstream aetiologies that assist in heart failure of ischaemic or non-ischaemic origin. Additionally, TMAO can induce cardiac hypertrophy and myocardial fibrosis in rats with aortic constriction, stimulate the increase of cardiac cell area and the expression of atrial natriuretic peptide and *β*-myosin heavy chain [55]. In addition, TMAO can activate NLRP3 (nucleotidebinding oligomerization domain–like receptor family pyrin domain-containing-3) inflammatory bodies to induce vascular inflammation through SIRT3-SOD2-mtROS (sirtuin-3-superoxide dismutase 2-mitochondrial reactive oxygen species) pathway [56], and also induce the expression of inflammatory genes in primary human aortic endothelial cells and vascular smooth muscle cells by activating nuclear factor(NF)-kB pathway [57]. TMAO can also up-regulate vascular cell adhesion molecule-1 expression, promoted monocyte adherence, activated protein kinase C and NF-kB [58]. These results demonstrate that TMAO may encourage the development of chronic heart failure by accelerating endothelial dysfunction, including decreasing endothelial self-repair and activating the inflammatory response. Studies in animal models have shown that TMAO pathway can directly lead to confrontational myocardial remodelling and the development of heart failure phenotypes. In the experiment of heart failure rats induced by transverse aortic arch constriction model, TMAO can promote ventricular remodelling, decrease left ventricular pump function, myocardial interstitial cells and perivascular fibrosis [59]. In mice fed a high choline diet, severe adverse ventricular remodelling and fibrosis were observed to be significantly increased, and the profibrotic TGF-β-Smads pathway was activated [60]. Savi and colleagues [61] observed that it worsened cardiomyocyte contractility in the presence of TMAO in vitro. Most importantly, the nature of the TMAO receptor remains unknown. TMAO is considered to act as a small molecule (a protein chaperone mimetic) that alters the conformation of the protein [62]. Therefore, TMAO may affect signalling pathways not only through classical receptor-ligand interactions but also as allosteric modifiers. TMA is observed through the GPCR trace amine-associated receptor 5 (TAAR5). TAAR5 has a high affinity with TMA but does not recognise TMAO [63]. Therefore, there is further optimism about the discovery of receptor-mediated TMAO effects.

### 4.4. The Gut Microbiota Metabolite-SCFA

SCFA signal via G-protein-coupled-receptors(GPCRS) such as GPR41, GPR43, and GPR109A and are essential regulators of gut homeostasis and epithelial barrier maintenance [64, 65]. Accumulated evidence indicates that SCFAs play a role in mediating the host immune system. For example, butyric acid regulates gene expression by inhibiting histone deacetylase (HDAC), thus increasing the number of Treg cells and enhancing their functions, to achieve anti-inflammatory purpose [66]. In addition, SCFAs could modulate host blood pressure. Propionate increases blood pressure by inducing renin secretion by binding to Olfr78, an olfactory receptor expressed in the glomerular paracycles of the kidney. However, propionate can also lower blood pressure by guaranteeing to GPR41 [15]. Moreover, SCFAs play a gut barrier-protective role. Butyrate can promote the proliferation and differentiation of intestinal epithelial cells, repair damaged intestinal mucosa, maintain the integrity of the intestinal mucosa, and reduce inflammation caused by exogenous substances such as bacteria and their metabolites entering the blood circulation [67]. SCFAs can also promote post infarction cardiac repair through inducing infiltration of CX3CR1+ monocytes in the peri-infarct zone [68]. In conclusion, SCFA can inhibit the occurrence and development of inflammation through multiple mechanisms, which are expected to improve the occurrence and development of heart failure.

### 4.5. The Gut Microbiota Metabolite-BAs

**Figure 1:**
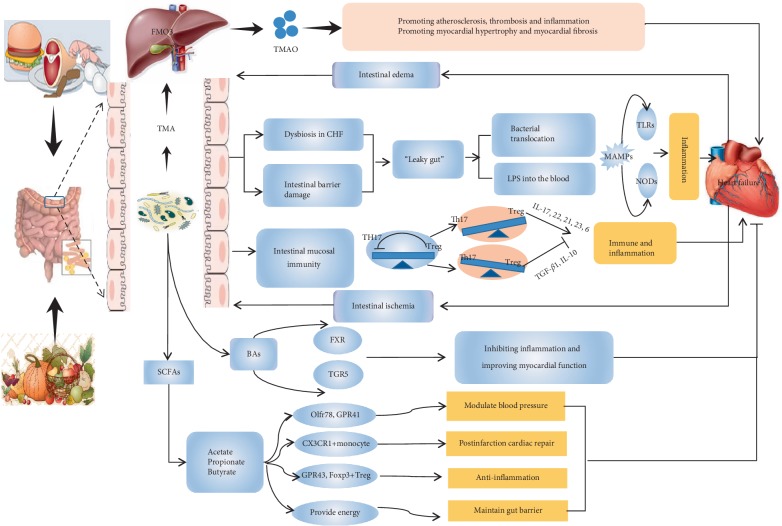
The role of gut microbiota in heart failure. TMA: Trimethylamine; TGF-*β*: transforming growth factor-*β*; TMAO: trimethylamine N-oxide; IL: interleukin; SCFA: short-chain fatty acids; LPS: lipopolysaccharide; FMO3: flavin monooxygenase-3; Treg: regulatory T cells; Th17: helper T cells 17; GPR: G protein receptor; MAMPs: microbe-associated molecular patterns; NOD: nucleotide oligomerization domain; FXR: farnesoid *X* receptor; TGR5: G-protein coupled bile acid receptor 1.

BAs are currently recognized as signalling molecules, and emerging evidence suggests that BAs affect cardiovascular function. The understanding of bile acid physiology was greatly expanded by the discovery of bile acid-responsive receptors, such as the farnesoid X receptor (known as FXR) and G-protein coupled bile acid receptor 1 (also known as TGR5). According to a cross-sectional study, an increased ratio of secondary to primary BAs in serum was found in patients with chronic heart failure, and this ratio was revealed to be associated with reduced overall survival in univariate analysis [69]. However, FXR can increase the imbalance of bile acid ratio, inhibit NF-kb, thereby reducing inflammation and improving myocardial function [70]. Bile acids, specifically TGR5 agonists, induce cytoprotective changes in the heart and improve myocardial response to physiologic, inotropic, and hemodynamic stress in mice [71]. Therefore, TGR5 agonists and FXR may be novel targets for the treatment of heart failure in the future (Figure 1).

### 4.6. Gut Microbiota and Hepatic Health

Numerous physiological processes in the body are accomplished through two-way interaction between the intestine and the liver. Through the portal vein system, intestinal flora transports various metabolic or immune substances, bacterial components or products to the liver [72], and the liver can also affect the intestinal function by secreting bile or immune factors, making the intestine and liver closely linked, known as the intestinal-hepatic axis. When intestinal flora is misregulated , the portal vein can be used as the channel between the intestinal tract and liver, so that endotoxin and peptidoglycan from intestinal tract continue to enter the liver. Many liver cells express innate immune cell receptors, such as toll-like receptors, which respond to intestinal microbial products and activate the immune response of the liver, leading to liver injury [73]. Current research shows that heart failure is closely connected with the liver and can lead to liver damage when heart failure occurs. We have previously known that enterotoxin metabolites leak into the systemic circulation during heart failure. Since the liver is the first organ to come into contact with toxic intestinal molecules, the interaction between the intestine and the liver during heart failure has become an exciting new field of research [74].

### 4.7. Gut Microbiota and Renal Health

Intestinal flora is closely related to the kidney. It was found that there were significant differences in the types and quantity of intestinal flora between patients with end-stage renal disease and healthy people [75]. In patients with chronic kidney disease, dominant intestinal flora can usually induce local or systemic inflammation, damage the intestinal mucosal barrier function and cause an inflammatory response, and translocate LPS and intestinal bacterial components into systemic blood circulation by increasing Th17/Treg ratio [76]. Cardiovascular diseases are closely linked to kidney diseases. In cardiorenal syndrome, heart-kidney interaction usually results in accelerated deterioration of two organs [77]. In the intestine, urea is hydrolysed by microorganisms to form a large amount of ammonia, which is then converted to ammonium hydroxide. Ammonia and ammonium hydroxide can destroy the tight junction of the intestinal epithelium, leading to the destruction of intestinal epithelial barrier function, make intestinal bacteria DNA and endotoxin into the systemic circulation, leading to the systemic inflammatory response [78]. Also, gut microbiota ferments tryptophan and tyrosine in food to generate uremic toxin indole sulfate and p-cresol sulfate. They can activate downstream mitogen-activated protein kinase (MAPK) and NF-kB by activating apoptotic signal regulator enzyme (ASK) −1, which mediates cardiac hypertrophy and cardiorenal fibrosis [79].

### 4.8. Gut Microbiota and Normal Cardiovascular Health

Typically, the intestinal flora is in balance and protects the cardiovascular system through metabolites. For example, enterolactone is produced primarily by intestinal digestion of fibre-rich foods.A study shows that a high serum enterolactone level is associated with reduced cardiovascular diseases mortality [80]. Protocatechuic acid is one of the main metabolites of complex polyphenols such as anthocyanins and procyanidins that are normally found in high concentrations in vegetables and fruit [81]. The studyconcluded that protocatechuic acid has an anti-atherosclerotic effect by promoting reverse cholesterol transport [82].

## 5. Gut Microbiota Interventions for Heart Failure

At present, the improvement or reversal of intestinal microflora has become a hot spot for heart failure, which contains the dietary intervention, prebiotic and probiotic therapy, faecal microbiota transplantation, antibiotic intervention, TMA-lyase inhibitors and so on.

### 5.1. Dietary Interventions

Diversity of gut microbiota was substantially correlated dietary habits, which confirmed the effect of long-term dietary patterns on gut microbiota. Studies have shown that adjusting diet for five days (short-term) can change the number and species of gut microbiota and produce corresponding changes to be adapted to dietary changes [83]. Dietary Approaches to Stop Hypertension (DASH) eating plan is a diet that is rich in fruit, vegetables, whole grains, and low-fat dairy foods. It includes meat, fish, poultry, nuts, and beans, and is bounded in sugar-sweetened foods and beverages, red meat, and added fats. The study has shown that the DASH diet can decrease the incidence of heart failure [84, 85]. Compared with patients receiving conventional heart failure management guidelines, patients receiving the DASH diet had better 6-minute walking test performance, quality of life, and tended to increase arterial elasticity after a 3-month intervention [86]. The Mediterranean diet refers to the eating styles of vegetables, fruit, fish, cereals, beans and olive oil in the southern European countries of the Mediterranean coast. This diet has been proven to prevent cardiovascular disease and reduce mortality from cardiovascular disease [87]. Studies have shown an increase in urinary TMAO levels in patients who do not comply with the Mediterranean diet [88]. Also, a high-fibre diet can improve the growth of acetate-producing bacteria, reduce blood pressure, and inhibit cardiac hypertrophy and fibrosis [89].

### 5.2. Prebiotic and Probiotic Therapy

Probiotics mainly include bifidobacteria, yeasts, lactic acid bacteria, and so on. They can inhibit inflammation, protect and repair intestinal mucosal barrier, and improve intestinal function. A study in rats showed that probiotics (*Lactobacillus rhamnosus *GR-1) could significantly improve left ventricular hypertrophy and ejection fraction in rats with acute myocardial infarction after six weeks of coronary artery occlusion [90]. What's more, probiotics can reduce myocardial cell apoptosis and improve ventricular remodelling in rats with spontaneous hypertension [91]. *Saccharomyces boulardii *can improve left atrial diameter and left ventricular ejection fraction in patients with chronic heart failure [92], and *Lactobacillus Plantarum 299V* can reduce infarct size and improve left ventricular function in rats [93]. However, probiotics remain at risk of probiotic translocation into the bloodstream and associated sepsis, and their safety needs additional study. Prebiotics is a dietary supplement, including isomalt oligosaccharides, oligosaccharides, Bifidus factors, and so on. It has a favourable effect on the host by selectively stimulating the growth and activity of bacteria. A recent study has shown that prebiotic oligofructose reduces infiltration of inflammatory cells in rats [94].

### 5.3. Fecal Microbiota Transplantation (FMT)

FMT is a method of treating intestinal microecological imbalance and reconstructing normal intestinal function by introducing bacteria or metabolites from donor faeces into diseased receptors. At present, FMT is mostly used to treat Clostridium difficile infection. Most of the treatment cases have few side effects, but the application of FMT to other diseases is still unknown [95]. Many studies are examining the effectiveness of FMT in the treatment of chronic diseases and have found that there may be super-donor phenomena, that is to say, faeces from specific donors are more likely to make FMT successful than those from other donors [96]. Clinical studies have shown that autologous faecal transplantation can quickly restore the gut microbiota diversity of healthy people after the use of antibiotics [97]. In a randomised double-blind controlled trial involving 20 patients with metabolic syndrome, it was found that faecal flora of vegetarians after single transplantation could change the intestinal flora structure of some patients, but could not change the parameters related to vasculitis [98]. Also, when faecal bacteria are transplanted, viruses are transplanted [99]. Therefore, FMT has both advantages and disadvantages in the treatment of diseases. How to balance it is still a problem to solve. For patients with heart failure risk factors or existing heart failure, it is possible to reduce TMAO by transplanting low-yield TMAO gut microbiota, but there is no such clinical study.

### 5.4. Antibiotics

The antibiotic treatment destroys the balance of intestinal flora, leading to the decrease of flora abundance and changes in composition. Studies have shown that NSAIDs can alter intestinal flora composition in elderly patients and have adverse effects [100]. Other studies have shown that when antibiotics are injected to eliminate intestinal bacterial translocation, it can alleviate systemic inflammation and myocardial cell damage in mice with myocardial infarction [101]. In addition to bactericidal and bacteriostasis, rifaximin can also reduce the toxicity and translocation of bacteria, has an anti-inflammatory effect and positively regulates the composition of intestinal flora [102], and promotes the growth of bifidobacteria and lactobacillus [103]. Polymyxin B and Tobramycin can reduce the LPS in the intestine and faeces, and the contents of IL-1*β*, IL-6 and TNF-*α* in vivo in patients with heart failure [104]. However, improper use of antibiotics can kill beneficial bacteria in the body, making pathogens resistant and causing various adverse reactions. Therefore, we should weigh the side effects of antibiotics and their clinical effects.

### 5.5. Microbial TMA-Lyase Inhibitors and Intestinal Mucosal Barrier Protectant

Some scholars have used choline analogues (compounds similar to choline) to inhibit the key enzyme CutC/D in the synthesis of TMA, thereby reducing the risk of cardiovascular disease by reducing the plasma TMAO level in mice [105]. Another recent study shows that resveratrol can stimulate the growth of beneficial bacteria in the intestinal tract through the reconstitution of intestinal microflora, thus decreasing the production of TMAO [103]. Urol is a metabolite of intestinal microflora derived from berries and pomegranate polyphenol. In vitro and mice, Urol and its synthetic analogue UAS03 can activate the pathways of aromatic hydrocarbon receptor (AhR) and nuclear factor red blood cell two related factor 2 (Nrf2) to enhance epithelial tight junction protein and enhance intestinal barrier function [106].

## 6. Conclusion

In conclusion, there are increasing shreds of evidence that gut microbiota disorders, intestinal barrier dysfunction and metabolites of gut microbiota are associated with heart failure. Intestinal barrier dysfunction and changes in the gut microbiota of composition may lead to abnormal production and absorption of gut microbiota metabolites in patients with heart failure. This imbalance can be expected to result in other complications such as heart dysfunction, inflammation, and so on. The composition of intestinal microflora in patients with heart failure is different from that in a healthy state. Reduction of SCFA-producing bacteria in patients with heart failure may be a noteworthy feature of patients with heart failure. In addition, the microbial potential for TMAO and LPS production increased significantly. More research has focused on the mechanism of microbial metabolites, and there is a need for clinical application of various therapeutic interventions. However, few studies have investigated in depth a direct role of the gut microbiota in heart failure and associated complications at the mechanistic and causal levels.Therefore, we need to further understand the role of gut microbiota in heart failure to better foster the development of diagnosis and treatment of heart failure.
